# Use of Biologic Extracellular Matrix in Two Ways to Reduce Cardiac Electronic Device Infection

**DOI:** 10.7759/cureus.13037

**Published:** 2021-01-31

**Authors:** Emily Buchanan, Dale Yoo

**Affiliations:** 1 Cardiac Electrophysiology, Heart Rhythm Specialists, PLLC, McKinney, USA

**Keywords:** device related infection, pacemaker complication, icd infection, extracellular matrix, extraction

## Abstract

Cardiac implantable electronic device (CIED) infections are a serious complication of both initial device implants and generator change procedures, and they are associated with a wide range of presentations. Reported rates of CIED infections vary widely from 0.1% to 19.9%, but it is estimated that they occur in 0.5% of initial device implants and 1-7% of subsequent implants. It is widely accepted that the administration of local antibiotics within the pocket as well as extracellular matrices (ECMs) can be utilized to reduce the incidence of CIED infections. We describe a case where the use of an additional biological ECM scaffold sutured directly into the incision site was utilized in addition to a biological ECM pouch in order to reduce the risk of infection. We propose that biological ECM could be utilized to reinforce the incision site directly as well as ECM within the pocket to reduce the instances of CIED infections. Further investigation of the use of biological ECM to prevent infection is warranted and paramount to further decrease the number of complications associated with device implantation.

## Introduction

Cardiac implantable electronic device (CIED) infections are a serious complication of both initial device implants and generator change procedures. They are associated with a wide range of presentations from cellulitis to pocket infections with subsequent sepsis [1]. Reported rates of CIED infections vary widely from 0.1% to 19.9%, but it is estimated that they occur in 0.5% of initial device implants and 1-7% of subsequent implants [2-5]. Of these infections, 60-80% are attributed to ﻿Staphylococcal species [6]. Comorbidities of CIED infections include renal dysfunction, heart failure, oral anticoagulation, and diabetes mellitus [7].

Treatment for CIED infections ranges from the administration of oral antibiotics to pocket revision procedures to laser lead extraction, depending on the location and severity of the infection. Although there has been some debate regarding the administration of long term antibiotics in lieu of pocket revision or device extraction, current guidelines recommend ﻿an aggressive approach of combined antimicrobial treatment and complete device removal [8]. The average cost of admissions due to CIED infections was determined to be ﻿$24,459 due to prolonged critical care stays and surgical interventions (pocket revision, device removal, or laser lead extraction) [7-9]. Treatment of CIED infection is especially challenging due to the development of a dense biofilm that surrounds the device. This biofilm prevents adequate penetration of antibiotics, thus proliferating bacterial growth [6]. Once this biofilm is formed, the entirety of the device must be removed, and the patient must undergo an intensive regimen of antibiotics before a new device can be implanted. Developing techniques that prevent initial device infection is paramount to preventing CIED infections and reducing the costs associated with this serious complication. In this report, we describe a case where the use of a biological extracellular matrix (ECM) in two ways, including it being sutured directly into the incision site, promoted incisional wound healing in a patient who had numerous CIED infections and suspected immunodeficiencies.

## Case presentation

The patient was a 75-year-old male with a history of coronary atherosclerosis, hypertension, and hyperlipidemia. He reported a prior history of sequential knee replacements with infections in each knee as well as a family history of immunoglobin deficiencies, which made him a very high risk for post-implantation infection. Initial implantation of a dual-chamber pacemaker was performed due to sick sinus syndrome. The device was placed in the left pectoral region without the utilization of a biological ECM pouch. At the initial two-week post-procedure wound check, erythema was observed at the incision with tenderness. Three weeks of oral antibiotics were administered, and the infection cleared without the removal of the device. A few years later, the patient developed congestive heart failure along with chronic right ventricular pacing, and the patient needed an upgrade of his dual-chamber pacemaker to a cardiac resynchronization therapy (CRT) system. This device was implanted using a biological ECM pouch due to the patient's history of prior infection.

Six weeks after the implantation of the CRT pacemaker, the patient was admitted to the hospital for a moderately sized hematoma and suspected infection of the device pocket and incision. Laser lead extraction of the complete device system was performed, and cultures obtained from the leads were determined to be acid-fast bacilli along with gram-negative rods. After a six-week regimen of broad-spectrum intravenous antibiotics, the patient underwent device reimplantation on the contralateral side. Due to concern regarding the patient's high risk of CIED infection within the pocket as well as at the incision site, a 10 cm by 1 cm strip of biological ECM, commonly used for vascular reconstruction, was utilized in addition to a biological ECM pouch enclosing the device. Both the strip and the pouch were hydrated in a gentamicin solution to further enhance the antimicrobial effects of the ECM, as described in the literature [10]. Instead of placing the additional biological ECM strip within the pocket, the strip was sewn directly into the incision site, where prior infections were observed (Video 1). Upon two-week, six-week, and six-month follow-up wound care visits, no erythema was observed. The patient report no fever, pain, or swelling at the incision site, and normal drainage was observed. We propose that, in this case, the utilization of biological ECM at the incision site in combination with a biological ECM pouch played a significant role in preventing infection in a patient with a high risk of infection.

**Video 1:** Implantation of Biological ECM Directly in the Device Incision Site

## Discussion

Initial techniques for reducing CIED infections involved the administration of antibiotic prophylaxis and placement of antibiotics inside the device pocket [11]. These methods were then improved by the development of biologic extracellular matrices (ECMs), which accelerate the wound healing process by providing scaffolding for new tissue and recruiting macrophages while simultaneously releasing antibiotics gradually into the device pocket [12-14]. Biological ECM scaffolding has been shown to promote extensive angiogenesis and constructive tissue remodeling via the recruitment of circulating progenitor cells [15]. In addition, early macrophage recruitment has been shown to improve the response to implanted materials and encourage tissue remodeling [8,16-18]. Biological ECMs are also designed to be gradually absorbed and replaced by subcutaneous tissue, thereby reducing chronic inflammation [13]. Together these properties have been shown to significantly reduce the instances of post-procedure infection [10,19].

It is widely accepted that administration of local antibiotics within the pocket as well as ECMs can be utilized to reduce the incidence of CIED infections [10,19]. This present case suggests that the use of an additional biological ECM scaffolding sutured directly into the incision site could play an important role in reducing CIED infections, especially in patients with immunodeficiency or a history of post-procedure infections. The utilization of a biological ECM strip at the incision site could play a role in accelerating wound closure by providing scaffolding for tissue remodeling that connects both sides of the incision. In addition, the extended-release of antibiotics may help fight bacterial invasion by providing local antibiotics directly to the incision site, where the risk of infection is significantly higher. We believe that biological ECM could be utilized to reinforce the incision site, thereby reducing the instances of CIED infections. 

## Conclusions

This report describes a case where the utilization of biological ECM at the incision site in combination with a biological ECM pouch played a significant role in preventing infection in a patient with a high risk of infection. This case contributes to the current efforts to reduce CIED infections through the novel application of biological ECM scaffolding sutured directly into the incision site where prior infections had been observed. Further investigation of the use of biological ECM to prevent infection is warranted and paramount to further decrease the number of complications associated with device implantation.
